# Single-cell transcriptomics reveals cell type–specific immune regulation associated with anti-NMDA receptor encephalitis in humans

**DOI:** 10.3389/fimmu.2022.1075675

**Published:** 2022-12-02

**Authors:** Yushu Jiang, Shuhua Dai, Linlin Jia, Lingzhi Qin, Milan Zhang, Huiqin Liu, Xiaojuan Wang, Rui Pang, Jiewen Zhang, Gongxin Peng, Wei Li

**Affiliations:** ^1^ Department of Neurology, Henan Joint International Research Laboratory of Accurate Diagnosis, Treatment, Research and Development, Henan Provincial People’s Hospital, People’s Hospital of Zhengzhou University, Zhengzhou, Henan, China; ^2^ Department of Neurology, Henan Provincial People’s Hospital, Xinxiang Medical University, Zhengzhou, Henan, China; ^3^ China Center for Bioinformatics, Institute of Basic Medical Sciences, Peking Union Medical College, Chinese Academy of Medical Sciences and School of Basic Medicine, Beijing, China

**Keywords:** anti-N-methyl-D-aspartate receptor encephalitis, peripheral blood mononuclear cell, single-cell RNA sequencing, B cells, plasma cells

## Abstract

**Introduction:**

Anti-N-methyl-D-aspartate receptor encephalitis (anti-NMDARE) is a rare autoimmune disease, and the peripheral immune characteristics associated with anti-NMDARE antibodies remain unclear.

**Methods:**

Herein, we characterized peripheral blood mononuclear cells from patients with anti-NMDARE and healthy individuals by single-cell RNA sequencing (scRNA-seq).

**Results:**

The transcriptional profiles of 129,217 cells were assessed, and 21 major cell clusters were identified. B-cell activation and differentiation, plasma cell expansion, and excessive inflammatory responses in innate immunity were all identified. Patients with anti-NMDARE showed higher expression levels of CXCL8, IL1B, IL6, TNF, TNFSF13, TNFSF13B, and NLRP3. We observed that anti-NMDARE patients in the acute phase expressed high levels of DC_CCR7 in human myeloid cells. Moreover, we observed that anti-NMDARE effects include oligoclonal expansions in response to immunizing agents. Strong humoral immunity and positive regulation of lymphocyte activation were observed in acute stage anti-NMDARE patients.

**Discussion:**

This high-dimensional single-cell profiling of the peripheral immune microenvironment suggests that potential mechanisms are involved in the pathogenesis and recovery of anti-NMDAREs.

## 1 Introduction

Anti-N-methyl-D-aspartate receptor encephalitis (anti-NMDARE) is a rare autoimmune disorder characterized by a complex neuropsychiatric condition (1) associated with antibodies against the glutamate receptor N1 (GluN1) subunit of NMDAR (2). Steroids and intravenous immunoglobulins (IVIG) are all used as first-line therapies for anti-NMDAREs (1, 3). However, approximately 40% of patients with anti-NMDARE showed no improvement after four weeks of first-line therapy (3). Due to the lack of disease awareness and late diagnosis, approximately 70% of anti-NMDARE patients are admitted to intensive care units with symptoms of persistent dysautonomia, consciousness fluctuation, or breathing dysfunction (4). Therefore, a thorough investigation of this disease is required to identify new markers and underlying mechanisms to cope with the severity of symptoms.

Susceptibility genes for anti-NMDARE include interferon regulatory factor 7 (IRF7), B cell scaffold with ankyrin repeats 1 (BANK1), T-Box Transcription Factor Protein 21 (TBX21), and human leukocyte antigen (HLA), as identified in genome-wide association studies (GWAS) (5–7). However, the association between these genes and disease susceptibility is weak; therefore, a thorough investigation is required to elucidate the exact mechanism. Tumors and herpes simplex encephalitis are recognized as the major causes of anti-NMDARE (8, 9), while molecular mimicry and chronic polyclonal expansions have been proposed as underlying pathogenic mechanisms (10, 11). Autoantibodies bind to and cross-link endogenous NMDARs, disturbing the interaction with the receptor tyrosine kinase EphB2, which leads to internalization and ultimately affects the function of NMDARs (12, 13). The reduced function of NMDARs results in learning and memory deficiencies and prominent psychiatric or behavioral symptoms in approximately 90% of patients (14). Some researchers believe that antibody titers can be used to make better clinical decisions; however, there is no reliable data to support this notion (15). Although its auto-antigen and effector mechanisms are well defined, the cellular and molecular mechanisms involved in anti-NMDARE are still poorly understood (1). Therefore, greater understanding of the activation and immune system patterns could provide important information regarding the pathogenesis of anti-NMDARE.

During the acute phase of anti-NMDARE, the levels of pro-inflammatory cytokines, such as interleukin (IL)-1β, IL-6, IL-17, and chemokines, such as CXCL-10, and CXCL-13 in the cerebrospinal fluid, and the pro-inflammatory cytokine IL-2 in plasma are elevated (16). Among these pro-inflammatory cytokines and chemokines, CXCL13 is involved in B cell-mediated neuroinflammation (17). B- cells differentiate into plasma cells and are involved in the production of anti-NMDAR-IgG and neuronal damage (18). The levels of the type 1 T helper (Th1) axis (IFN-γ, TNF-α, CCL3, and CXCL10), Th2 axis (CCL1, CCL8, CCL17, CCL22), Treg axis (IL-10), Th17 axis (IL-7), B cell axis (CXCL13), cytokines, and T cells also contribute to the clinical stages of the disease (16, 19, 20). The immune system comprises a vast variety of cells in different states; however, previous studies conducting immunophenotypic analysis were based on low-flux assays confined to selected cell types and markers (21, 22). Therefore, a better understanding of immune system modulation in response to anti-NMDARE using high-flux assays is required.

In the current study, we performed scRNA-seq of peripheral blood mononuclear cells (PBMC) using supervised and unsupervised machine-learning tools to dissect immune dysregulation in anti-NMDARE. This analysis identified 21 major cell groups, allowing us to assess the primary alterations in these major cell types. We found that patients with anti-NMDARE disease expressed high levels of IL-1B, IL-6, IL-8, TNF, CXCL8, TNFSF13B, TNFSF13, and NLRP3, whereas in the acute phase, high levels of DC_CCR7 were expressed in human myeloid cells. Moreover, in the acute phase of disease in patients with anti-NMDARE antibodies, strong humoral immunity and positive regulation of lymphocyte activation had developed. This high-dimensional single-cell profile of the peripheral immune microenvironment suggests that several potential mechanisms are involved in the pathogenesis and recovery of anti-NMDAREs. Moreover, we observed that, compared to HCs, anti-NMDARE patients produced elevated levels of pro-inflammatory cytokines and chemokines. The present study was designed to obtain a better understanding of the heterogeneity within the immune system related to anti-NMDARE through high-flux assays, predominantly single-cell RNA sequencing (scRNA-seq).

## 2 Materials and methods

### 2.1 Processing of patient samples

This study was approved by the Ethics Committee of Henan Provincial People’s Hospital. 34 suspected anti-NMDARE patients were enrolled in the Neurology Department of Henan Provincial Peoples Hospital between December 2020 and January 2022.

The inclusion criteria for patients with anti-NMDARE were as follows: (1) diagnosis of anti-NMDARE according to the Graus and Dalmau criteria (23); (2) in the cerebrospinal fluid (CSF), antibody titers of more than 1:100–1:320 were considered for anti-NMDARE. The exclusion criteria were as follows: (1) definite or suspected central nervous system infection; (2) definite or suspected peripheral infection; (3) definite or suspected neuromyelitis optica spectrum disorders or multiple sclerosis (MS); (4) definite or suspected systemic immune disease; (5) history of malignant tumor; (6) pregnancy; and (7) history of high-dose methylprednisolone pulse, intravenous immunoglobulin, or plasma exchange treatment. Finally, 10 patients were included in the study cohort (**Supplementary Figure 1**). In addition, five age- and sex- matched HCs were enrolled. The first cohort, including anti-NMDARE (n = 5) and HCs (n = 5), was used for 10X genomics scRNA-sEq. The second cohort, including anti-NMDARE (n = 10) and HCs (n = 5), was used for multiple microsphere flow immunofluorescence analysis (**Supplementary Table 1**). Ten patients with anti-NMDARE received high-dose methylprednisolone pulse therapy and intravenous immunoglobulin. Peripheral blood was collected the day before and ten days after the onset of first-line therapy. Informed consent was obtained from all patients and HCs.

### 2.2 Generation and sequencing of single-cell libraries

Fresh blood samples were diluted in phosphate- buffered saline (PBS), and the PBMC fraction was isolated using SepMate 50 tubes (Stemcell Technologies) and human Lymphocyte Separation Medium (Cedarlane). The cell pellets were resuspended at 2-4 × 10^6^ cells/mL in serum-free, animal protein-free cell freezing medium, immediately cryopreserved at −80° Celsius for no more than one week. Then they were transferred to liquid nitrogen storage before further processing. After resuscitation of frozen aliquots, cell viability was assessed using 0.4% trypan blue (Thermo Fisher, Cat. no. 14190144) on a Countess^®^ II Automated Cell Counter (Thermo Fisher Scientific). ScRNA-seq libraries were constructed with the 5’ Library and Gel Bead Kit and V(D)J Enrichment Kit and prepared per the Chromium Single Cell 5’ library preparation kit user guide (10X Genomics). 10X library preparation and sequencing Beads with a unique molecular identifier (UMI) and cell barcodes were loaded close to saturation so that each cell was paired with a bead in a gel bead in emulsion (GEM). After exposure to the cell lysis buffer, polyadenylated RNA molecules were hybridized to the beads. Beads were retrieved in a single tube for reverse transcription. For cDNA synthesis, each cDNA molecule was tagged on the 5’end (corresponding to the 3’ end of a messenger RNA transcript) with UMI and a cell label indicating its cell of origin. Subsequently, 10X beads were subjected to second-strand cDNA synthesis, adaptor ligation, and universal amplification. Sequencing libraries were prepared using randomly interrupted whole-transcriptome amplification products to enrich the 3’ end of transcripts linked to the cell barcode and UMI. All remaining procedures, including library construction, were performed in accordance with the manufacturer’s protocol (CG000206 RevD). ScRNA-Seq libraries were sequenced on NovaSeq6000 (Illumina) with paired-end 150bp sequencing.

### 2.3 The V (d) J library preparation and sequencing

Individual cells were encapsulated together with gel beads with a bar code and primer inside an oil droplet using a microfluidics system. Subsequently, the gel beads within each oil droplet were dissolved, and the cells were split to release mRNA. The mRNA was reverse transcribed into cDNA using the 10X barcode and UMI. After breaking the emulsions, the cDNA was split into two parts for gene expression and library construction. The V (d) J sequences of T-cell receptor (TCR) and B cell receptor (BCR) were amplified by PCR using nested primers designed for region C. Due to the mRNA information retaining the 5’-ends of reads, unlike the 10X Genomics 3’mRNA library, the sequencing then allowed accessing to the large amounts of single-cell gene expression and immune-group library data.

### 2.4 ScRNA-seq bioinformatics analysis

#### 2.4.1 Construction, quality control, and filtering of feature-barcode matrix

During initial procession 10X data “mkfastq” module of the Cell Ranger (5.0.0) pipeline was operated to demultiplex the Illumina raw base call files (BCLs) obtained from Illumina sequencing into FASTQ files. FASTQs generated from the above workflow subsequently underwent several processing steps, as shown below. Specifically, according to default and recommended parameters based on the Cellranger “count” module (https://support.10Xgenomics.com/single-cell-gene-expression/software/pipelines/latest/using/count), the FASTQ sequences were aligned and quantified to the GRCh38 1.2.0 human reference genome obtained from 10X Genomics. The outputs generated by multiple runs of the Cellranger “count” were aggregated into a new feature-barcode matrix (barcodes, features, and count matrix) using the “aggr” pipeline. The matrix from the above operations was then imputed into the Seurat R package (4.1.3) for quality control and downstream analyses. To exclude low-quality cells from all samples, we adopted three criteria to remove genes detected in less than three cells, cells with less than 200 or over 6000 expressed genes, and cells expressing >0.1 of mitochondrial genes. After filtering, 116,916 cells with a median gene count of 18827 genes were maintained for the subsequent analysis, as shown in **Supplementary Table 2**. Finally, we corrected the batch effects between samples using the R package Seurat prior to clustering.

#### 2.4.2 Feature selection, dimension reduction, and visualization for high-dimensional data

The “LongNormalize” method was used for normalization based on the filtered gene-barcode matrices obtained in the previous step. The “MVP” method was used to identify 494 highly variable genes (HVGs). We retained only the genes that contributed to group variability after controlling for the strong relationship between variability and average expression. Next, data from different samples were identified as ‘anchors’ and integrated. FindIntegrationAnchors and IntegrateData in the Seurat package were used to obtain pairs of cell anchor points when the cells of the query data set and the cells of the reference data set had common molecular characteristics. We subsequently performed principal component analysis (PCA) for linear dimension reduction and reduced the data to the top 20 PCs based on the elbow plot after scaling. Clustering was further performed using the Seurat FindClusters function with a resolution of 0.5, and the clusters were visualized on a 2D map produced with uniform manifold approximation and projection (UMAP). The final 12 cell clusters were determined using the above method. For sub-clustering, we applied the same procedure of scaling, dimensionality reduction, and clustering to a specific set of data (usually restricted to one type of cell).

#### 2.4.3 Identification of differentially expressed genes (DEGs) and marker genes

Specific marker genes for each cluster were calculated using the Seurat “FindAllMarkers” function, with the parameters: logfc.threshold > 0.25, min.diff.pct > 0.25, min.pct > 0.1. DEGs were then identified by comparing cells from the target cluster to all other cells from the remaining clusters using Wilcoxon rank-sum tests. Finally, according to the statistical test results, the genes with the highest ranking from DEGs were set as specific marker genes of that cluster based on LFC > 0.5 and P-value < 0.05. Furthermore, marker genes were simultaneously defined as those with the highest mean expression in that cluster.

#### 2.4.4 Functional enrichment analysis of DEGs

The ClusterRprofile R package (v4.2.2) was used to perform Gene Ontology (GO) analysis and Kyoto Encyclopedia of Genes and Genomes (KEGG) pathway analysis.

#### 2.4.5 Pseudo time-trajectory analysis of different cell types

We used DDRTree’s reduction method in Monocle3 (R package) with default parameters to reconstruct pseudo- time trajectories of ‘target cells’ to predict cellular differentiation pathways. First, we filtered all the differences of the cell clusters after converting normalized data to a Monocle object in R, and reduced the dimension for building minimum generating trees. Second, we searched for the optimal sorting of single -cell data in high-dimensional and low-dimensional spaces. Finally, we fitted the pseudo- time trajectory curve for the best cell development or differentiation. The criteria applied for gene selection included the following analysis: Genes expressed in less than 10 cells, or with a minimum normalized expression greater than 0.1 were filtered out, 2) q-value<0.01 in DEGs expression analysis.

#### 2.4.6 BCR and TCR data analysis

The reads of a single barcode from the scRNA-seq were put into the vdj pipeline from the Cell Ranger V(D)J pipeline (v3.1.0, 10X Genomics), and these reads were glued to assemble a set of contigs to produce the best estimation of the current transcriptional sequence. The purpose of V(D)J contig annotation was to compare V, D, and J fragments to a contig; thus, we identified complementary determining region 3 (CDR3) sequences and rearranged full-length BCR/TCR V(D)J segments, as well as clonotype frequency. Next, the following sequences were obtained for downstream analysis: 1) high- confidence, detectable V genes, J genes, CDR3 nucleotides, and 2) more than two unique molecular identifier (UMI) counts. In cases with more than one assembled heavy-light chain pair, the one with higher UMI counts was chosen as the dominant in the corresponding cells. T or B cells that shared the same CDR3 nucleotide sequence of the VJ and VDJ chains were considered to be one clonotype that has the same adaptive immune receptor and epitopes. The Sc-BCR/Sc-TCR data were analyzed using the R package scRepertoire v1.5.2., following to the official vignette.

#### 2.4.7 Statistical analysis

Shapiro-Wilk normality test and Levene’s test were used to determining the normality and variance homogeneity. The wilcox-test was used for statistical analysis to determine the genes expression regulation change among multiple cell clusters. All statistical analyses were performed using the open-source statistical package R version 4.0.3 (R Project for Statistical Computing, Vienna, Austria).

### 2.5 Cytokine and chemokine measurement

Several cytokines and chemokines, including IL-1β, IL-2, IL-4, IL-5, IL-6, IL-8, IL-10, IL-17, IFN-α, IFN-γ, and TNF-α, were detected by multiple microsphere flow immunofluorescence. Cytokine detection reagent was provided by Qingdao Raisecare Biotechnology Co., Ltd. (Shandong, China). Briefly, EDTA-K2 anti-coagulated whole blood was centrifuged at 1,000 g for 30 min, and plasma was collected. Subsequently, 25 µL each of experimental buffer, centrifuged plasma, capture microsphere antibody, and detection antibodies were mixed in flow tubes and placed on a shaker at 500 RPM for 2 h at room temperature. Then, 25 µL of streptavidin-phycoerythrin (SA-PE) was added to the flow tubes, which were then placed on a shaker at 500 RPM for an additional 30 min. Data were then obtained using an automatic flow cytometer (Raisecare).

## 3 Results

### 3.1 Study design and single-cell survey of major changes in transcriptional profiles between anti-NMDARE patients and HCs

Ten fresh peripheral blood samples were obtained from five patients with anti-NMDARE (P1-P5), in whom the diagnosis of anti-NMDARE was based on the Graus and Dalmau criteria (23). Blood samples were obtained twice from each patient, once the day before the onset of first-line therapy (steroids and intravenous immunoglobulins) and once ten days later. Peripheral blood samples were collected from five age- and sex- matched healthy donors as controls (HC1-HC5). The clinical characteristics of the patients are presented in **Supplementary Table 3**.

**Figure 1:**
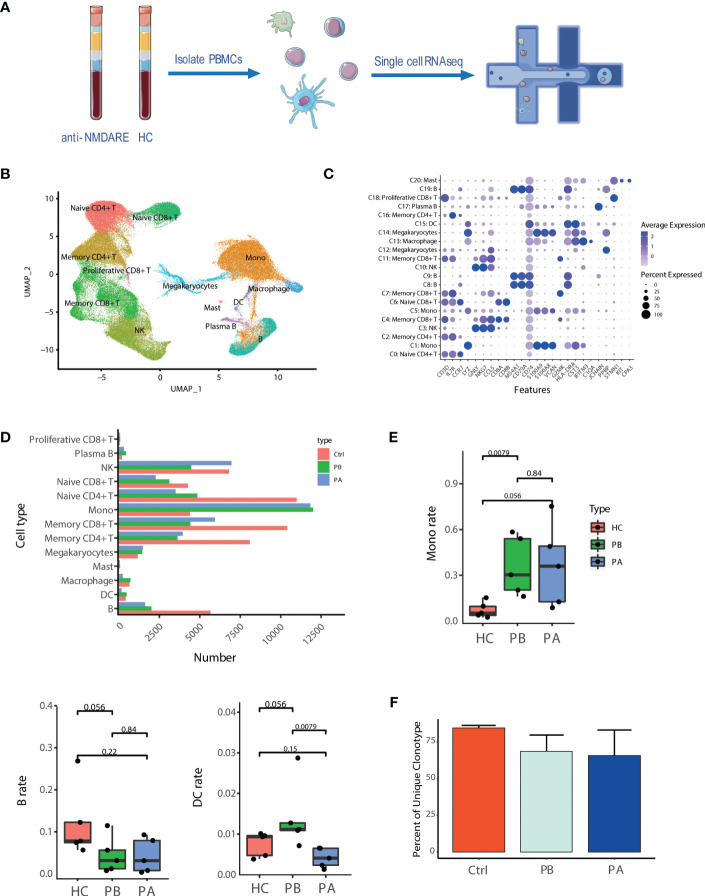
Assessment of major changes in transcriptional profiles in anti-NMDARE patients and HCs: **(A)** Schematic representation of scRNA-seq analysis; **(B)** Uniform manifold approximation and projection (UMAP) representation of scRNA-seq data; **(C)** Cluster annotation of expression values of selected genes (x axis) across each cluster (y axis) was shown by dot plot represents; **(D)** The alignment of the number of each cell type was shown by histogram across HCs (n = 5), PB (n = 5) and PA (n = 5); **(E)** The proportions of the major immune cell types among HCs, PBs and Pas; **(F)** Percent of unique clonotype across PBs, PAs and HCs.

A total of 129,217 cells were analyzed by scRNA-seq using the 10X genomics sequencing technology. Among these, 37,696 were derived from patients prior to first-line therapies (PBs), 38,396 after therapy (PAs), and 53,125 from HCs (**Figure 1A**). The sequencing information of each sample is presented in **Supplementary Table 2**. The single-cell profiles were divided into 21 clusters (**Supplementary Figure 2**), of which the major cell types included CD4+T cells, CD8+ T cells, B cells, monocytes, natural killer (NK) cells, macrophages (MΦ), mast cells (MCs), dendritic cells (DCs), and residual megakaryocytes (Mgk) mixed in PBMCs (**Figure 1B**). Most marker genes of each cluster were calculated based on highly DEGs (**Supplementary Table 4**). The cell types were identified using known unique signature and marker genes. Subsequently, 13 cell types were generated: naïve CD4+ T cells, memory CD4+ T cells, naïve CD8+ T cells, memory CD8+ T cells, proliferative CD8+ T cells, B cells, plasma B cells, NK cells, monocytes, MΦ, MCs, DCs, and Mgk (**Figure 1C**). Cluster annotation was confirmed by gene set enrichment analysis. GO analysis (**Supplementary Figure 3**) demonstrated that clusters 8, 9, and 17 were associated with B- cell receptor signaling; c lusters 0, 2, 4, 6, 7,11 and 16 were associated with T- cell activation and differentiation; clusters 1, 5, and 13 were found to regulate cytokine production and myeloid cell activation and differentiation, and c luster 15 was associated with antigen processing and presentation. These results were consistent with the KEGG pathway analysis (**Supplementary Figure 4**).

Compared to HCs, anti-NMDAR patients demonstrated a notable difference in the amount of cell subtypes (**Figure 1D** and **Supplementary Figure 5**). Compared with HCs, pre-treatment anti-NMDARE patients showed a higher proportion of monocytes and DCs, and a lower proportion of B cells and T cells. (**Figure 1E** and **Supplementary Figure 6**). Moreover, we assessed differences in subtype representations between PBs and PAs, finding that the population of B cells and myeloid cells demonstrated a decrease in PAs and DCs, while MΦ, in terms of myeloid cells, demonstrated a prominently lower population than HCs (**Figures 1D, E**, **Supplementary Figure 6**).

BCR and TCR information was retained by single-cell BCR sequencing (scBCR-seq) and single-cell TCR sequencing (scTCR-seq), based on scRNA-seq libraries. After quality control, 9,992 and 46,420 cells were detected with BCR (IGH) and TCR (TCR α–β pair) signatures, respectively (**Supplementary Tables 5, 6**). Among them, 8,817 B cells with a single productive IGH allele (1,941 from PBs, 1,899 from PAs, and 4,977 from HCs) and 41,475 T cells with a single productive TCRα-β pair (11,691 from PBs, 8,860 from PAs, and 20,924 from HCs) were detected. In the remainder of the study, we focused predominantly on B cells because of their importance in anti-NMDARE (**Figures 1D, E**). The proportion of unique clonal BCRs of HCs, PBs and PAs is 84.49%, 68.61% and 65.67%, respectively (**Figure 1F**). A significant decrease in proportion of unique clonal BCRs was observed in all anti-NMDARE patients compared to HCs. And this decrease continued after the first-line therapy. The large and hyperexpanded categories is uncommon in HCs, according to **Supplementary Figure 7**. The large and hyperexpanded categories is significantly taken into consideration in the PBs and PAs, particularly in P1a_PA and P4b_PB. The relative abundance of large and hyperexpanded categories demonstrated an increase in anti-NMDAR patients group compared to HC group. Our observations suggested that anti-NMDARE comprises oligoclonal expansions in response to immunizing antigens. Overall, the major immune cells and characterized cell proportions in anti-NMDARE patients were identified and compared to HCs, revealing prominent changes in B cells and myeloid cells.

### 3.2 Anti-NMDARE induces strong humoral immune responses

**Figure 2:**
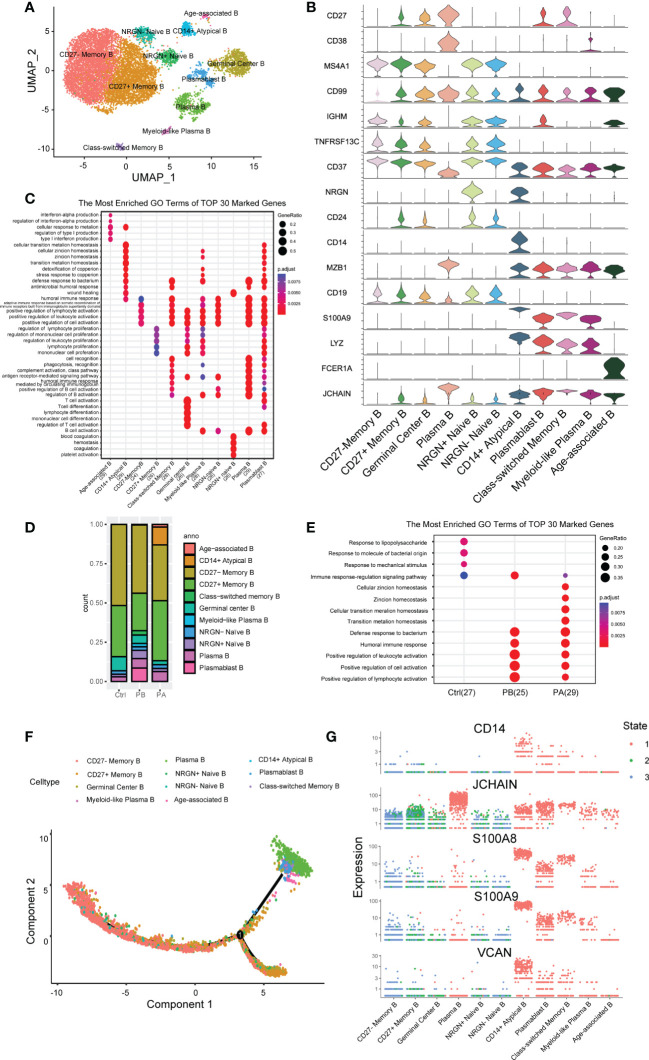
Assessment of changes in B cells in transcriptional profiles in anti-NMDARE patients and HCs: **(A)** The heterogeneous clusters of B cells were shown by UMAP; **(B)** The key gene markers across B cell subsets were shown by Violin plots; **(C)** The DEGs in B cell subsets; **(D)** Percentage of B cell types under each condition; **(E)** Enrichment analysis of DEGs from B cells in PBs, PAs and HCs; **(F)** B cells sorted using the DDRTree algorithm and projected onto the different cell states; **(G)** Key genes related to differentiation in B cell subsets across each state.

B cells were further divided into more homogeneous subsets, and 11 distinct sub-clusters were obtained (**Figure 2A**). Given the reported marker genes (24, 25), NRGN+ naive B cells (cluster 4), NRGN- naive B cells (cluster 5), germinal center B cells (cluster 2), CD27- memory B cells (cluster 0), CD27+ memory B cells (cluster 1), class-switched memory B cells (cluster 8), Plasmablast B cells (cluster 7), Plasma B cells (cluster 3), myeloid-like plasma B cells (cluster 9), CD14+ atypical B cells (cluster 6), and Age-associated B cells (cluster 10) were identified (**Figure 2B**).

Differential analysis revealed that naive B cells expressed the marker genes IGHD, IGHM, and CD37, but very low levels of CD27. Memory B cells (MBCs) expressed very low levels of IGHD. Unbiased analysis revealed two sub-clusters of naive B cells: NRGN^high^ and NRGN^low^ B cells. Neurogranin, encoded by NRGN, bidirectionally modulates synaptic plasticity *via* the calmodulin-dependent regulation of the neuronal phosphoproteome (26). Additionally, three sub-clusters of MBCs were identified. Cluster 8 was identified as class-switched memory B cells with upregulated CD99 and low IGHM levels (27). CD27 memory B cells are increased in the elderly and in patients with specific autoimmune diseases (28). Germinal center B Cells had a higher expression of TNFRSF13C, and persistent germinal center activity may be responsible for the ongoing production of NR1-IgG, which strongly contributes to the initial peripheral generation of NR1 antibodies (29, 30). Antibody-secreting cells (ASCs) comprising plasmablasts, plasma cells, and myeloid-like plasma B cells expressed higher CD27 and CD38, concurrent with the low expression of CD19 and MS4A1. One cluster of ITGAX^high^TBX21^high^IGHD^low^CD27^low^CD24^low^ B cells was identified as age-associated B cells (ABCs), which have attracted significant attention in recent years (31). The high expression of FCER1A in age-associated B cells suggests increased antigen-presenting activity. ABCs may be potential triggers for autoimmune diseases (32). CD14+ atypical B- cells, characterized by the absence of CD21 and CD27, mainly exist in PAs. MT2A, MT1G, MTIX, and MTIE are predominantly expressed by CD14+ atypical B cells, which affect apoptotic and autophagy pathways in various diseases (33).

Furthermore, we characterized the transcriptomic changes in B cells of all clusters (**Figure 2C**, **Supplementary Tables 7 and 8**). GO analysis revealed that cluster 0 was associated with positive regulation of lymphocyte activation, cytokines, toll-like receptors (TLRs), and B- cell receptor signaling pathways. Conversely, cluster 1 was associated with regulation of lymphocyte proliferation, positive regulation of cell adhesion, antigen processing, and presentation of peptide antigens. Cluster 3 was associated with intrinsic signaling, including protein maturation, ERAD pathway, and complement activation. Cluster 6 was associated with phagocytosis, cell chemotaxis, and cell migration. Cluster 10 was associated with ATP metabolic processes and material transport. KEGG analysis further showed that clusters 3 and 7 shared similar pathways, such as the cell cycle set, indicating that they were highly activated.

### 3.3 Extensive B cell heterogeneity

The B cell compartments differed greatly in composition across the different cell groups (**Figure 2D**, **Supplementary Figure 8**). Compared with HCs, PBs showed an increased proportion of class-switched memory B Cells and ASCs, along with a decreased proportion of MBCs and germinal center B cells. These proportional changes indicate a strong humoral immune response induced by anti-NMDARE. After first-line therapy, the proportion of ASCs and class-switched memory B cells in total B cells decreased with an increase in ABCs and CD14+ Atypical B cells. In summary, strong humoral immunity develops in patients with anti-NMDARE in the acute stage. Considering that ASCs were significantly expanded in PAs and demonstrated essential roles at producing high-affinity antibodies, we were able to transcriptional changes between anti-NMDARE patients and HCs.

GO analysis suggested that the DEGs were mainly involved in the positive regulation of lymphocyte activation and humoral immune response (**Figure 2E**). Genes involved in lymphocyte activation, that is, IGHA1, IGHG1, IGHV3-30, IL7R, CCL5, TYROBP, LGALS1, and IGKC, were upregulated in anti-NMDARE patients compared to HCs. Genes upregulated in the humoral immune response in PBs compared to HCs included S100A4, S100A9, S100A8, S100A12, LYZ, GNLY, IGHV3-23, and JCHAIN. After therapy, genes involved in the response to steroids and those involved in the apoptotic process of leukocytes were induced, including TSC22D3, DDIT4, GPX1, and TXNIP. In general, immune response signaling and lymphocyte activation pathways were activated in PBs, whereas apoptotic signaling pathways were upregulated in PAs.

Further, we analyzed the trajectory of B cells in each sample (**Figure 2F**, **Supplementary Figure 9**). In HCs, trajectory analysis revealed a gradual transition from naïve B cells and germinal center B cells to ASCs or MBCs, with most cells undergoing differentiation into MBCs. MBCs had low progression along pseudonyms, indicating a close relationship to naïve B cells, which marked the beginning of the pseudotime. In anti-NMDARE patients, although the differentiation pathways were similar, more cells differentiated into ASCs. After therapy, the end of the branch generated ABCs and CD14+ Atypical B cells, which were closely associated with the immune senescence, including reduced B cell genesis and a dampened immune responses (34). The branch consisting of ASCs, ABCs, CD14+ Atypical B, and Class-switched memory B cells progressed further along pseudotime, showing that these cell populations were differentiated further away from naive B cells compared to MBCs. ASCs highly expressed JCHAIN, which is required for immunoglobulin polymerization (**Figure 2G**), J chain is a small glycopeptide linked to IgA and IgM by disulfide bonds which has also been detected in IgG- and IgD-containing cells (35, 36). Clusters 6 and 8 contained elevated levels of S100A8 and S100A9, which are considered alarming and damage-associated molecules, respectively (37). The above analysis revealed an enhanced propensity for differentiation towards the ASC phenotype in anti-NMDARE patients, and a highly activated state of ABCs and CD14+ Atypical B cells.

### 3.4 Characterization of BCRs

**Figure 3:**
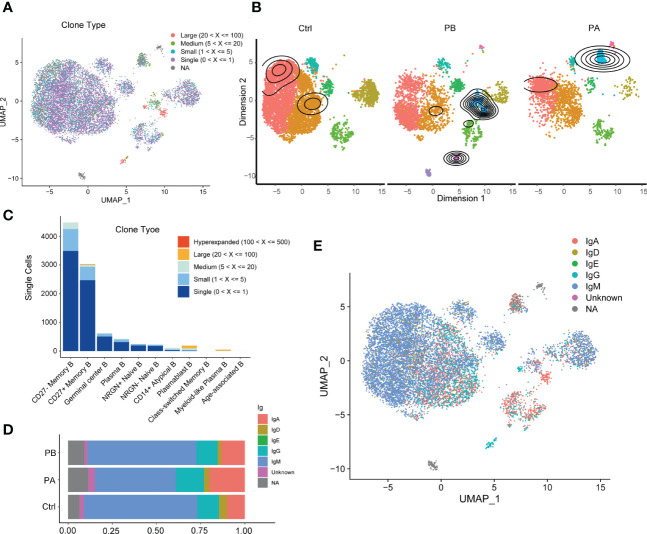
Characterization of BCR repertoires during acute anti-NMDARE state: **(A)** The clonal expansion status in B cells shown by UMAP; **(B)** The clonal expansion status of B cell subsets in PBs, PAs and HCs; **(C)** BCR clonotype tracking in B cell subsets; **(D)** The percentage of IGHA and IGHG in BCRs across PBs, PAs and HCs; **(E)** The IGH isotypes in each B cluster shown by UMAP.

The dynamics of BCR repertoires during acute anti-NMDARE were dissected (**Figure 3A**). The PBs of clonally expanded B cells showed transcriptional homogeneity; however, a diffusive distribution indicated transcriptional heterogeneity in the HCs (**Figure 3B**). Moreover, the number of clonal BCRs increased significantly in plasma B cells and myeloid-like plasma B cells (**Figure 3C**). Prior to undergoing first-line therapies, the majority of clone types from plasmablast B cells and myeloid-like plasma B cells were large (5<X<=100), but exhibited various expression patterns in PAs. However, after first-line therapy, the proportion of IGHA and IGHG increased significantly (**Figure 3D**), indicating that B cell activation led to the conversion of immunoglobulin from IgM/IgD to IgG/IgA. Significant oligoclonal expansion in ASCs was observed, among which the lgG subtype was predominantly found in myeloid-like plasma B cells and IgA in plasma and plasmablast B cells (**Figure 3E**, **Supplementary Figure 10**). In general, our data suggested that a larger proportion of ASCs with highly expanded features and transcriptional homogeneity were present in patients with anti-NMDARE antibodies, and that significant oligoclonal expansions were dominated by IgA and IgG isotypes.

### 3.5 Expression of inflammatory cytokines and chemokines in myeloid subsets

**Figure 4:**
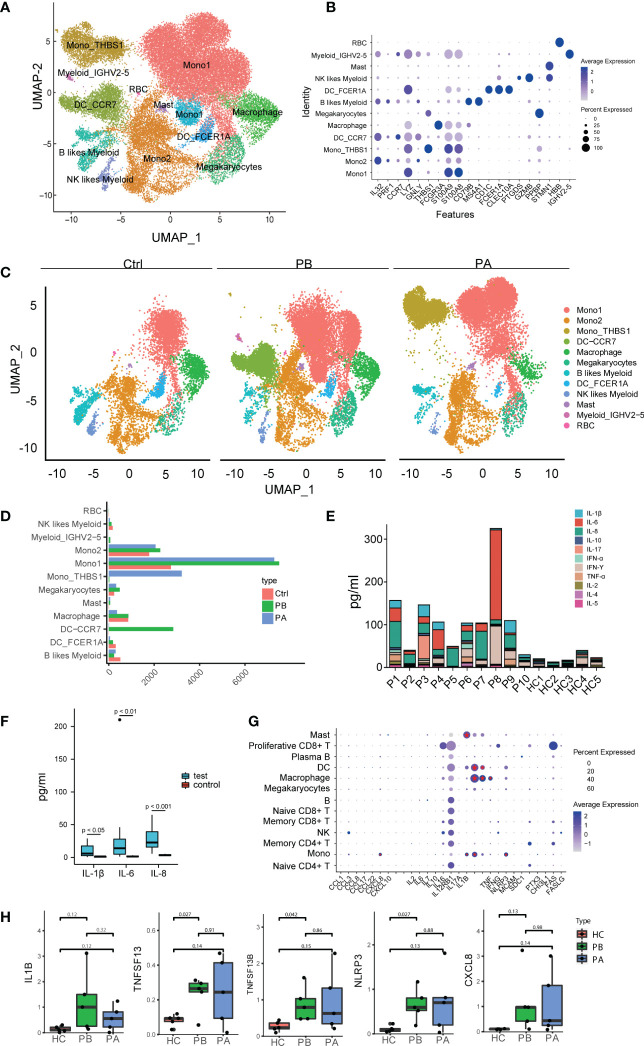
Assessment of changes in myeloid cells in transcriptional profiles between anti-NMDARE patients and HCs: **(A)** The heterogeneous clusters of myeloid cells were shown by UMAP; **(B)** The key gene markers across myeloid cell subsets were shown by violin plots. The dot plot represents the expression values of selected genes (x axis) across each cluster (y axis). The percentage of cells expressing the marker of interest were shown by dot size and the mean expression within expressing cells was shown by color intensity. **(C)** The major myeloid subsets from the HC, PB, and PA groups were shown by UMAP; **(D)** Cell abundance across PBs, PAs and HCs; **(E)** The alignment of the levels of selected cytokines and chemokines in peripheral blood in HCs (n = 5), PBs (n = 10); **(F)** The levels of IL-1β, IL-6 and IL-8 in peripheral blood in HCs (n = 5), PBs (n = 10). P-value was adjusted by false discovery rate; **(G)** Expression patterns of molecular biomarkers in anti-NMDARE. The dot plot represented average expression levels and percentage of cells expressing the molecular biomarkers genes across peripheral PBMC; **(H)** Expression levels of IL1B, TNFSF13B, TNFSF13 and NLRP3 in PBMCs of HCs (n = 5), PBs (n = 5) and PAs (n = 5).

Furthermore, we quantitatively evaluated the anti-NMDARE-driven changes in myeloid cells. Human peripheral blood myeloid cells, including monocytes, DCs, and MΦ, promote antigen presentation and inflammatory activity (38). Myeloid cells were sub-grouped into 13 clusters, numbered from 0 to 12 (**Figures 4A, B**). The three myeloid subsets were defined as monocytes (clusters 0, 1, and 2), of which Mono1 (Cluster 0) was the most abundant. The expression levels of S100A8, S100A9, and LYZ were higher in mono1 (cluster 0). In mono2 cells (cluster 1), the expression levels of IL-32, PRF1, and GNLY were higher. M ono _THBS1 (cluster 2) was a minor subset expressing THBS1. The myeloid subset MΦ (cluster 4) highly expressed FCGR3A (cluster 4). Myeloid_IGHV2-5 (cluster 11) highly expressed IGHV2-5 (cluster 11). Mgk (cluster 5) and MCs (cluster 10) highly expressed surface markers STMN1 and PPBP. A few cells, defined as B cells, NK cells, and red blood cells were combined with myeloid cells (clusters 6, 9, and 12). The myeloid subsets DC_FCER1A (cluster 8) and DC_CCR7 (cluster 3) highly expressed FCER1A and CCR7, respectively (**Figures 4A, B**).

We subsequently compared the expression levels of different myeloid clusters (**Figure 4C**). In both mono1 and mono2, monocyte expansion was prominent (**Figure 4D**). After first- line therapy, mono_THBS1 was highly expressed in patients. The proportion of mono_THBS1 remained significantly higher in patients with anti-NMDARE post-treatment than in HCs. Mono1 showed high expression levels of S100A8 and S100A9, which exert pro-inflammatory effects in a range of diseases (39). Mono2 highly expresses IL32 and PRF1. IL-32 may further play a role in innate and adaptive immune responses, which induce other cytokines involved in inflammation, including tumor necrosis factor (TNF)-α, IL-6, and IL-1β, and activate typical cytokine signaling pathways of NF-κB and p38 MAPK (40). PRF1 is important for immunity (41). Thus, anti-NMDARE patients showing an inflammatory state were caused by the expansion of mono1 and mono2. Moreover, we observed that during the acute phase in anti-NMDARE patients, higher levels of DC_CCR7 were expressed in human myeloid cells (**Figure 4C**). The composition of mono1 and mono2 in the PB and PA groups were similar, suggesting that immunological changes were sustained during convalescence. However, the increased proportion of mono_THBS1, along with a decreased proportion of DC_CCR7 in PAs suggests that excessive inflammatory responses were inhibited.

Furthermore, the levels of several cytokines and chemokines in the peripheral blood of the anti-NMDARE patients were measured (**Figure 4E**, **Supplementary Table 1**). Compared with HCs, anti-NMDARE patients had much higher levels of IL-1β, IL-6, and IL-8 inflammatory cytokines (**Figure 4F**, **Supplementary Figure 11**). The expression of molecular biomarkers in patients with anti-NMDARE was also measured (**Figure 4G**). IL-6 and IL-2 are crucial inflammatory mediators that stimulate both B and T cells in autoimmune processes, and have been used as therapeutic targets to treat anti-NMDAREs (42, 43). Overall, we found that IL6 was predominantly expressed in B cells, while CXCL8, IL1B, TNFSF13B, TNFSF13, TNF, and NLRP3 were mainly expressed in myeloid cells and upregulated in PBs (**Figures 4G, H**). In addition, CXCL8 and NLRP3 were mainly expressed in monocytes. TNFSF13 and TNF were mainly expressed in macrophages, whereas TNFSF13B was mainly expressed in monocytes, DCs, and macrophages (**Supplementary Figure 12**). These results suggest that as myeloid abundance increases, the production of total chemokines and other molecular biomarkers in anti-NMDARE patients is expected to increase.

## 4 Discussion

Anti-NMDARE is the most frequently recognized neuronal antibody-mediated encephalitis (44). ASCs enter the central nervous system through the blood-brain barrier and secrete antibodies, leading to their internalization and subsequently reduced functioning of NMDARE, delineating the pathogenesis of the disease (45, 46). The immune system is dysregulated in anti-NMDARE patients, and significant efforts have been made in the research and treatment of anti-NMDARE (47). However, further investigation of the regulation of the immune system in peripheral blood is still required to develop novel therapies and to achieve improved curative effects in less time.

In the current study, we aimed to understand the cellular transcriptional changes in anti-NMDARE patients. To the best of our knowledge, this is the first study to create a high-resolution map of transcriptional changes and systematically discuss cellular heterogeneity and impaired peripheral tolerance in anti-NMDARE patients.

We performed scRNA-seq to assess the transcriptional profiles of 129,217 cells and identified and annotated 21 major cell clusters, after which we performed by DEG and pathway analysis. First, we identified 21 cell clusters comprising CD4+T cells, CD8+ T cells, B cells, monocytes, NK cells, MΦ, MCs, Mgk, and DCs. B cell differentiation and activation, plasma cell expansion, and excessive inflammatory responses in innate immunity were all identified. Furthermore, we found some interesting results: anti-NMDARE promotes B-cell polarization from naive to ASC with higher CD27 and CD38 expression, and low expression of CD19 and MS4A1, and anti-NMDARE induces a dysregulation in the balance of myeloid subsets (e.g., a significant increase in mono1, mono2, and DC _CCR7). NMDAR are taken up by antigen- presenting cells (APCs), and present to the immune system, resulting in the differentiation of naive B cells into MBCs, and plasma cells in local lymph nodes (48, 49). Based on these two studies, we speculated that in patients with anti-NMDARE, naive B cells differentiate into more plasma cells with the help of APCs. Previous studies of pathology in anti-NMDARE have focused on adaptive immunity (50), finding that monocytes and DCs were significantly expanded in PBs compared to HCs; this suggests that, both innate and adaptive immunity are involved in the occurrence and development of diseases.

In the current study, we identified DEGs and observed remarkable oligoclonal expansions in anti-NMDARE patients, which may provide a reference for studying the pathological roles of immune cell subsets and discovering potential drug targets to treat these diseases.

B cells are the dominant cell type for maintaining humoral defenses (51). They are also associated with the development and control of autoimmunity that targets self-antigens (52, 53). Various B cell categories exhibit the ability for both self-protection and self-destruction (52). Patients with anti-NMDARE carry major B cell alterations, including expansion of the activated plasma B cell phenotype (54), with higher concentrations of NR1-IgG in the serum than the CSF (55, 56). These alterations suggest that the periphery is likely the site of primary immunization. We found that although the total B cell abundance decreased in anti-NMDARE patients, the proportion of class-switched memory B cells and ASCs among the B cells increased.

Moreover, we observed extensive oligoclonal expansion of BCRs and isotype switching from IgM/IgD to IgG/IgA. All the evidence supports the hypothesis that due to some specific antigens, the acquired immune responses thus generated leads to anti-NMDARE (13, 57). B rain biopsy or autopsy findings suggested that during anti-NMDARE, B cells and plasma cells infiltrate brain tissue along with IgG deposits, resulting in little neuron loss (58, 59), which confirmed that adaptive immunity is involved in the occurrence of diseases. The detection of synthetic antibodies based on oligoclonal IgAs and lgGs can be used as a specific diagnostic test for anti-NMDARE (47, 60, 61). The DEG analysis in our study indicated that genes involved in positive regulation of lymphocytes and humoral immune responses such as IL-7R, CCL5, LYZ, GNLY, JCHAIN, and S100 were upregulated. The expression levels of IGHA1, IGHG1, IGHV3-30, and IGHV3-23 encoding immunoglobulin heavy chains were high before first-line therapy and returned to normal levels after therapy, suggesting that the immunomodulatory effect of steroids and IVIG may be related to the blockage of activated immunoglobulin (62, 63). In autoimmune disorders, the expansion of ABCs and CD14+ Atypical B cells is significantly higher (64, 65); therefore, conditional targeting of the transcription factor T-bet encoded by TBX21, which is important and sufficient for ABCs formation (66), could be an efficient and novel therapeutic target.

Previous studies have focused on adaptive immunity in anti-NMDARE (1); however, we observed remarkable expansion and alterations in the differentiation of myeloid cells. In our study, mono1 and mono2 highly expressed S100A8, S100A9, IL32, and PRF1, indicating an inflammatory state in patients with anti-NMDARE. S100A8 and S100A9 can also amplify the inflammatory response by promoting the secretion of pro-inflammatory cytokines (TNF, IL6, etc.), and exert a chemo-attractive function that allows the recruitment and adhesion of leukocytes (67).

Our data showed a dysregulation in the balance of myeloid populations in anti-NMDARE patients, as manifested by a substantial increase in monocyte subsets and DC_CCR7. The central chemokine receptor CCR7 plays a role in T-cell activation, differentiation, and expansion of IgG-producing B cells (68). Blocking CCR7 signaling seems to reduce both humoral and immune cell-mediated pathogenic courses, making CCR7 a potential target for interference with anti-NMDARE. Thus, we suggest CCR7 as a possible target for potential future drugs with an antagonistic effect in inhibiting disease progression by reducing inflammation (69).

Furthermore, we found an increased percentage of mono_THBS1, a potent inhibitor of T cell and DC activation which dampens an excessive inflammatory response (70, 71), after first-line therapy, Therefore, we propose that mono_THBS1 could be used as an indicator to monitor the curative effect. However, the increased proportion of mono_THBS1 and the decreased proportion of DC_CCR7 in PAs suggests that excessive inflammatory responses were inhibited.

Patients with anti-NMDARE have higher levels of IL-1β, IL-6, IL-8 and higher expression of inflammatory factors, particularly CXCL8, IL1B, TNFSF13B, TNFSF13, TNF, and NLRP3, than in HCs. Our data suggest that myeloid cells in patients with anti-NMDARE may contribute to local inflammation, and cytokine storms are associated with disease severity. Moreover, several studies have confirmed that some cytokines/chemokines and other molecular biomarkers, such as the NLRP3 inflammasome, soluble Fas and FasL, chitinase-3-like 1 (CHI3L1), pentraxin 3 (PTX3), and CD40L, are associated with clinical activity, inflammation, and long-term outcomes (72).

However, our study has several limitations. The sample size for scRNA-seq analysis was small and heterogeneous. This study was carried out on PBMCs, and therefore could not reflect the inflammatory responses in the cerebrospinal fluid. F actors such as age, disease severity, and immunoregulatory therapies were not comprehensively assessed. Moreover, more patients with herpes simplex encephalitis or teratoma should be included in future studies to accurately determine the relationship between different infections and immune responses.

## Data availability statement

The RNA-seq data reported in this article has been deposited in the Genome Sequence Archive (GSA) for human in BIG Data Center, Beijing Institute of Genomics, Chinese Academy of Sciences and is accessible through accession number HRA003508.

## Ethics statement

This study was approved by the ethics committee of Henan Provincial Peoples Hospital. Written informed consent to participate in this study was provided by the participants’ legal guardian/next of kin.

## Author contributions

YJ played a major role in the design of the research, conduction of the experiments, acquisition and analysis of the data, drafting, and revision of the manuscript. SD was involved in the sample collection, conduction of the experiments and analysis of the data. LJ, XW, and RP collected the sample. GP analyzed the data. LQ, MZ, and HL edited and revised manuscript. WL and JZ co-directed the study. WL revised manuscript and provided financial support. All authors contributed to the article and approved the submitted version.

## Funding

This work was supported by grant to WL from the Medical Science and Technology Project of Henan Province (NO. SBGJ2018077). The funder had no role in study design, data collection and analysis, decision to publish, or preparation of the manuscript.

## Acknowledgments

**Figure 1A** was constructed in part using Servier Medical Art (https://smart.servier.com/), licensed under a Creative Common Attribution 3.0 Generic License. (https://creativecommons.org/licenses/by/3.0/).

## Conflict of interest

The authors declare that the research was conducted in the absence of any commercial or financial relationships that could be construed as a potential conflict of interest.

## Publisher’s note

All claims expressed in this article are solely those of the authors and do not necessarily represent those of their affiliated organizations, or those of the publisher, the editors and the reviewers. Any product that may be evaluated in this article, or claim that may be made by its manufacturer, is not guaranteed or endorsed by the publisher.

## Abbreviations

anti-NMDARE, anti-N-methyl-D-aspartate receptor encephalitis; ASCs, antibody-secreting cells; BANK1, B cell scaffold with ankyrin repeats 1; BCR, B cell receptor; BCLs, base call files; CSF, cerebrospinal fluid; CDR3, complementary determining region 3; CHI3L1, chitinase-3-like 1; DEGs, differentially expressed genes; DCs, dendritic cells; GluN1, glutamate receptor N1; GWAS, genome-wide association studies; GEM, gel bead in emulsion; GO, Gene Ontology; HLA, human leukocyte antigen; HCs, healthy controls; HVGs, highly variable genes; IVIG, intravenous immunoglobulins; IRF7, interferon regulatory factor 7; IL, interleukin; KEGG, Kyoto Encyclopedia of Genes and Genomes; MΦ, macrophages; MCs, mast cells; Mgk, megakaryocytes; MBCs, memory B cells; NK, natural killer; PBMC, peripheral blood mononuclear cell; PBS, phosphate-buffered saline; PCA, principal component analysis; PBs, patients prior to first-line therapies; PAs, patients after first-line therapies; PTX3, pentraxin 3; scRNA-seq, single-cell RNA sequencing; scBCR-seq, single-cell BCR sequencing; scTCR-seq, single-cell TCR sequencing; SA-PE, streptavidin-phycoerythrin; TBX21, T-Box Transcription Factor Protein 21; Th1, type 1 T helper; TCR, T-cell receptor; TLRs, toll-like receptors; TNF, tumor necrosis factor; UMI, unique molecular identifier; UMAP, uniform manifold approximation and projection.
