# Supplementary material for: Single-cell transcriptomics reveals cell type–specific immune regulation associated with anti-NMDA receptor encephalitis in humans
Source: Front Immunol. 2022 Dec 2;13:1075675. doi: 10.3389/fimmu.2022.1075675 (PMC9762154; doi:10.3389/fimmu.2022.1075675)
Supplement: Supplementary file 1 [file DataSheet_1.docx]

***Supplementary Material***

# Supplementary Figures and Tables

# 1.1 Supplementary Figures


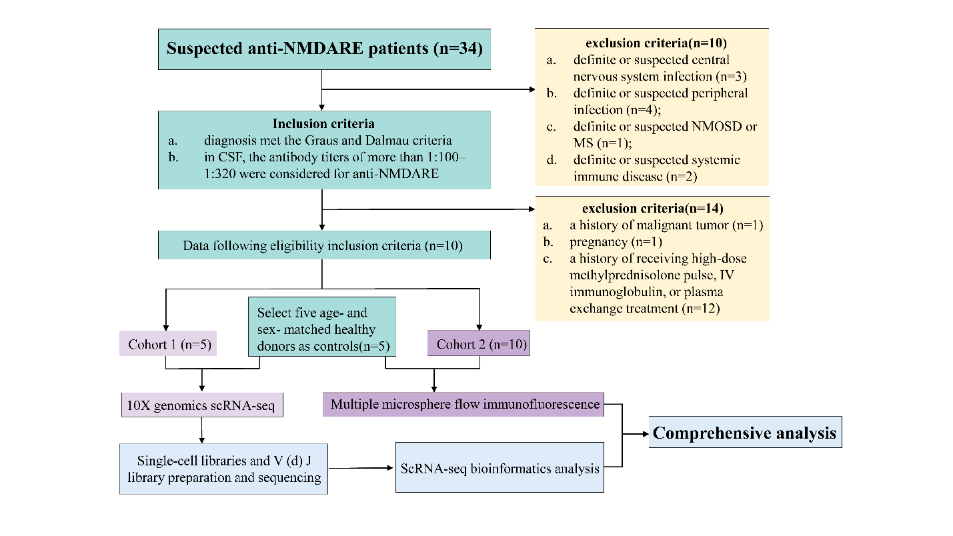


**Supplemental Figure 1.** Flow diagram of the enrollment of patients with anti-NMDARE patients.


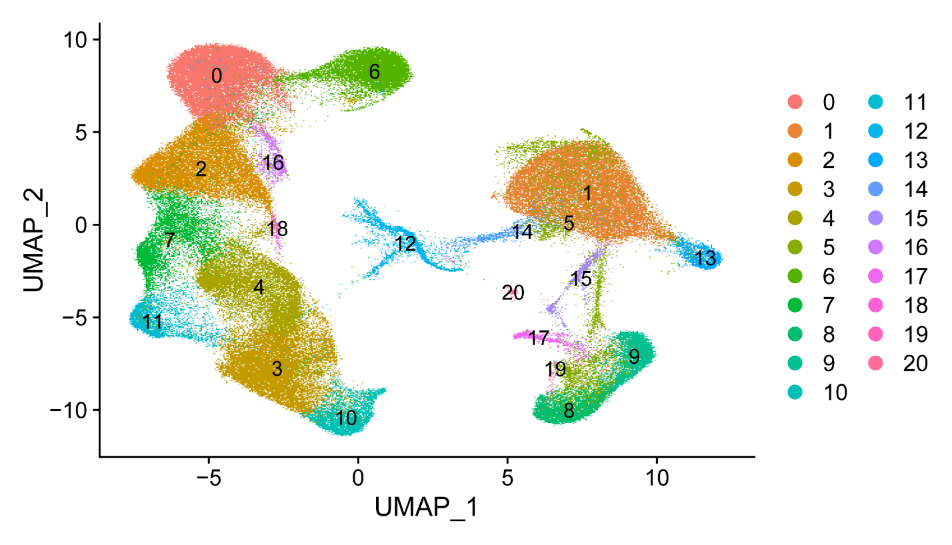


**Supplemental Figure 2.** A UMAP plot representing the 21 clusters across 129,217 PBMCs from 5 anti-NMDARE patients before and after first-line therapies and 5 HCs.


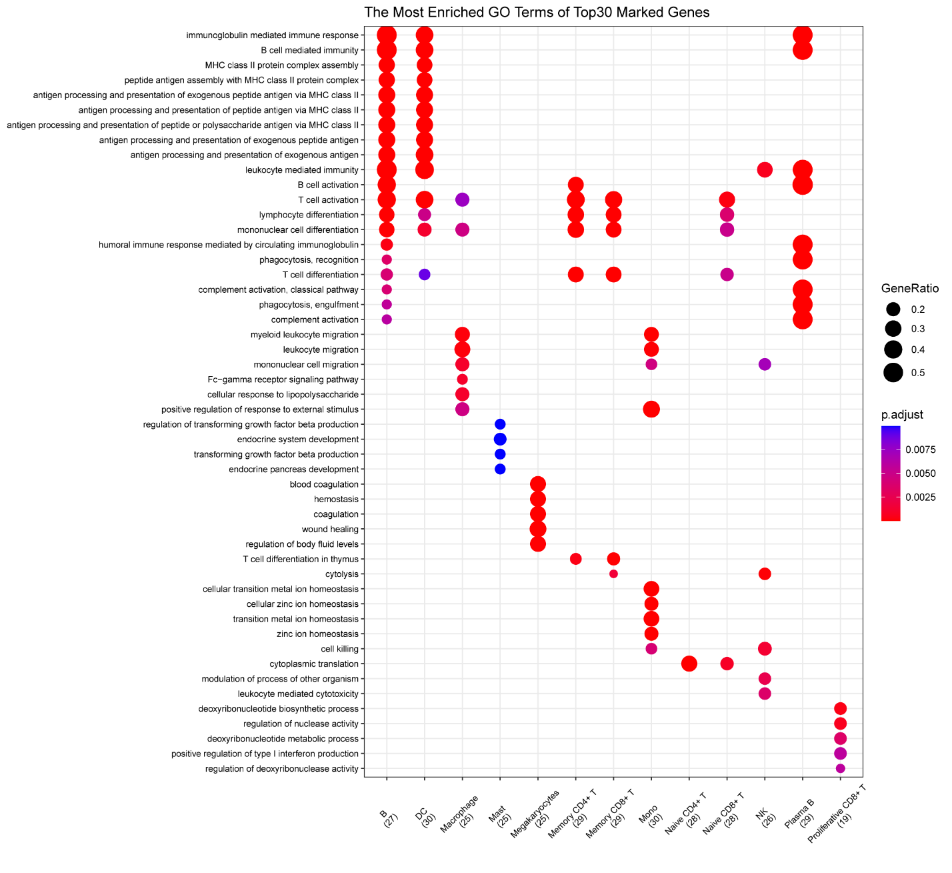


**Supplemental Figure 3.** GO enrichment analysis for major cell types.


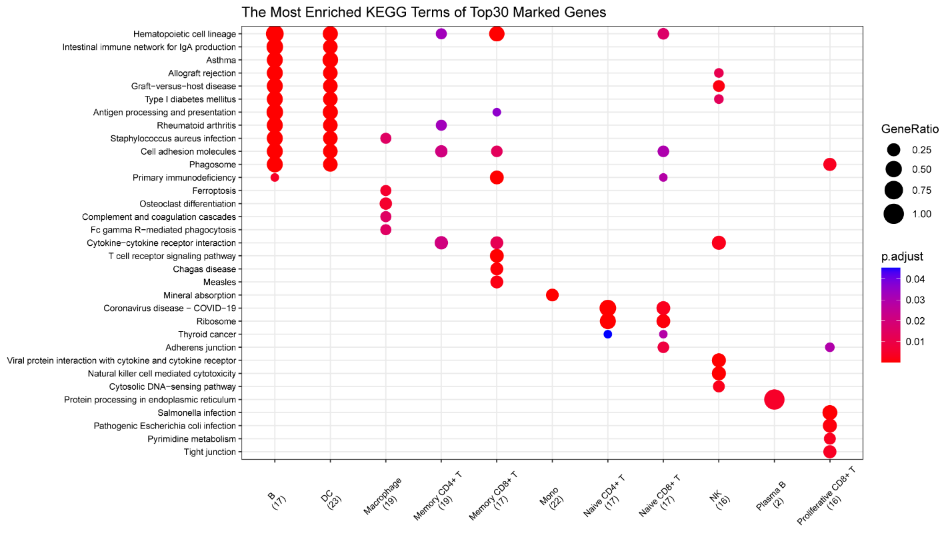


**Supplemental Figure 4.** KEGG enrichment analysis for major cell types.


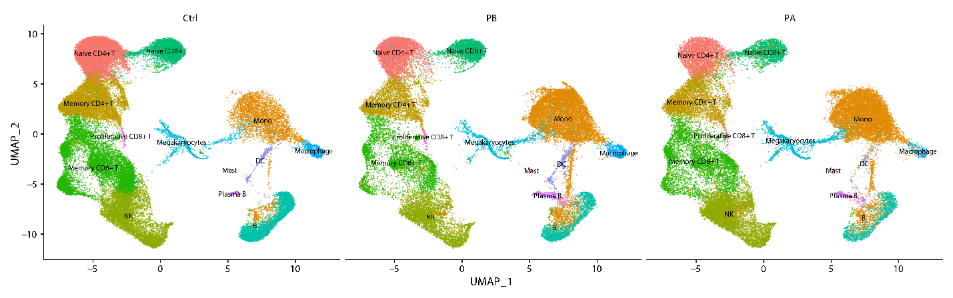


**Supplemental Figure 5.** The alignment of PBMC cell clusters by UMAP across HCs (n = 5), PBs (n = 5) and PAs (n = 5).


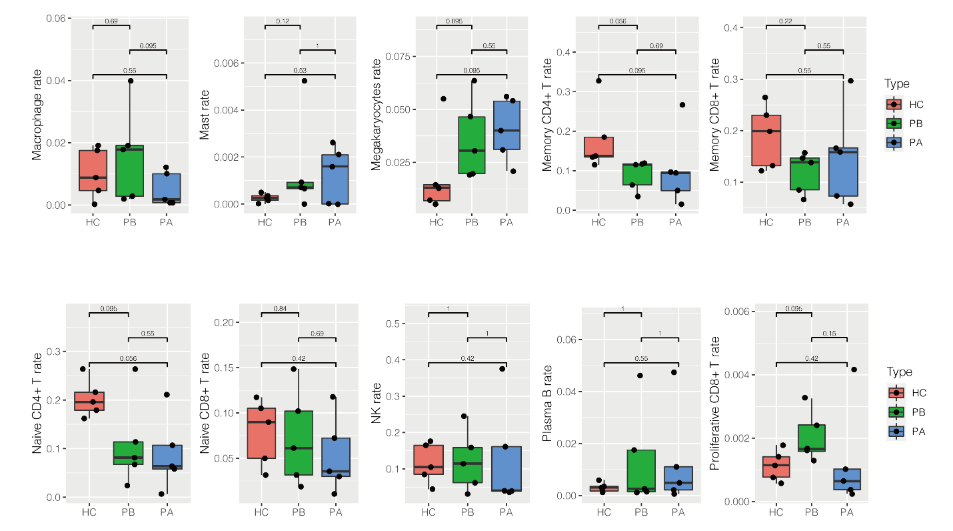


**Supplemental Figure 6.** The proportions of the major immune cell types among HCs, PBs and PAs (using wilcox-test for pairwise comparisons).


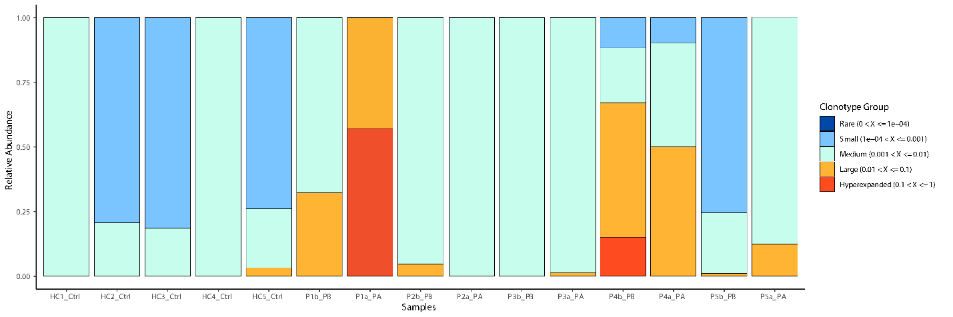


**Supplemental Figure 7.** The relative abundance of different gene clonetypes in the whole clone space. This analysis divided the clonetypes into five categories: rare, small, medium, large and hyperexpanded.


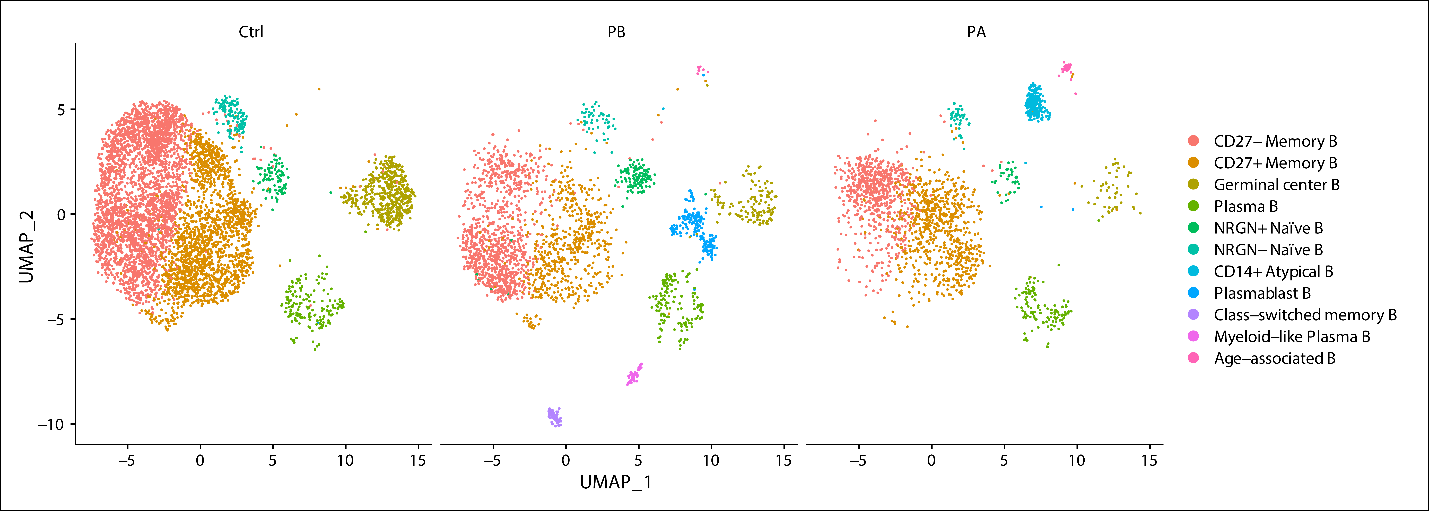


**Supplemental Figure 8.** UMAP projection of 11 B cell groups among controls (n = 5) and patients (PB, n = 5; PA, n = 5).

**Supplemental Figure 9.** Distribution of pseudotime among controls (n = 5) and patients (PB, n = 5; PA, n = 5).


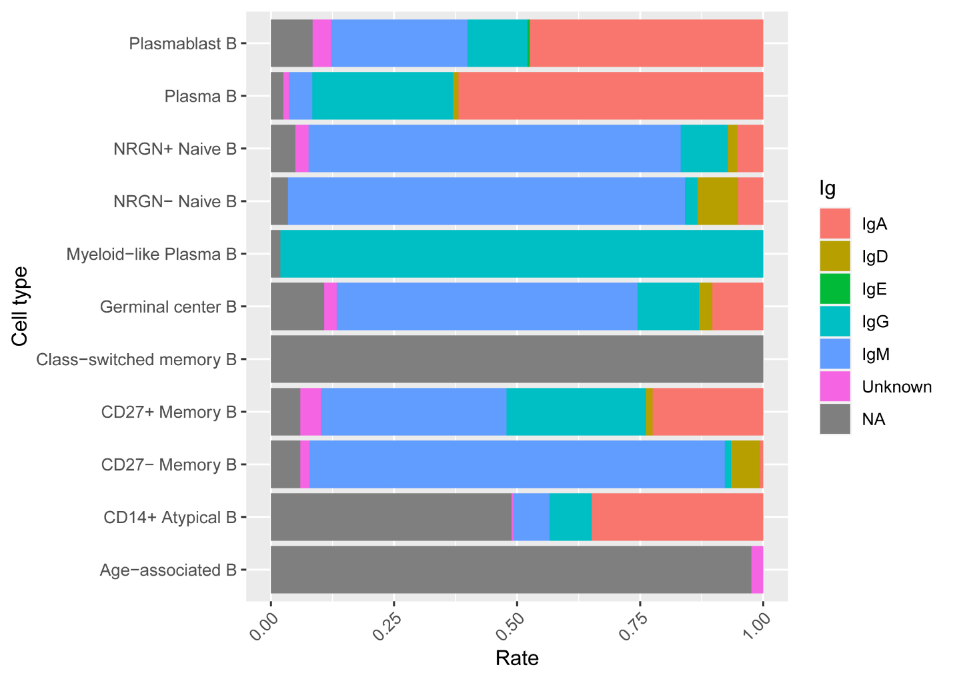


**Supplemental Figure 10.** Proportion of IGH isotypes in each B cluster.


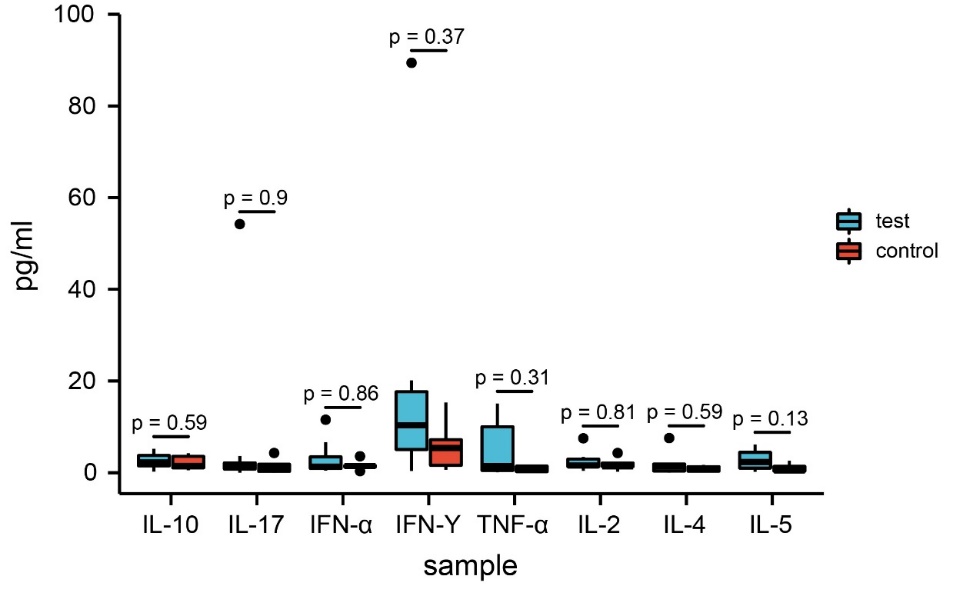


**Supplemental Figure 11.** The levels of selected cytokines and chemokines in peripheral blood across HCs (n = 5), PBs (n = 10).


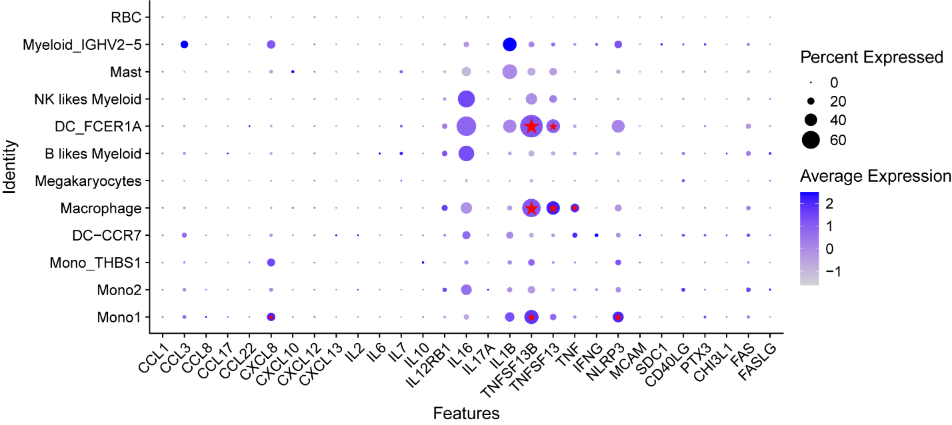


**Supplemental Figure 12.** Expression patterns of molecular biomarkers in myeloid cells.

- 1. **Supplementary Tables**

**Supplemental table 1** Demographic characteristics of study populations

| Category | Cohort 1 | | Cohort 2 | |
| --- | --- | --- | --- | --- |
|  | anti-NMDARE | HC | anti-NMDARE | HC |
| Age, mean (SD) | 29.80±17.33 | 24.50±6.40 | 27.9±14.46 | 24.50±6.40 |
| Female, n (%) | 2(40) | 3(60) | 5(50) | 3(60) |
| Conscious disturbance | 3(60%) | 0 | 5(50%) | 0 |
| Seizure | 2(40%) | 0 | 4(40%) | 0 |
| Memory deficit | 2(40%) | 0 | 4(40%) | 0 |
| Psychiatric symptoms | 2(40%) | 0 | 5(50%) | 0 |
| Movement disorders | 1(20%) | 0 | 3(30%) | 0 |
| Antibody positivity in cerebrospinal fluid | 5(100%) | 0 | 10(100%) | 0 |
| Antibody positivity in serum | 2(40%) | 0 | 5(50%) | 0 |

Anti-NMDARE, anti-N-methyl-D-aspartate receptor encephalitis; HC, healthy control;

# Supplemental table 3 Clinical features of subjects in first cohort of the study

| **Patient** | **Age (years)** | **Sex** | **Onset to immunotherapy (days)** | **Initial symptoms** | **symptoms at 10 days after the onset of first-line therapies** | **CSF** | | | | **MRI** |
| --- | --- | --- | --- | --- | --- | --- | --- | --- | --- | --- |
|  |  |  |  |  |  | **Leukocyte (*10^6/L)** | **Chloride (mmol/L)** | **Glucose (mmol/L)** | **Protein (g/L)** |  |
| 1 | 18 | female | 13 | Conscious disturbance, seizure, psychiatric symptoms | Psychiatric symptoms | 19 | 133 | 3.42 | 0.3 | Normal |
| 2 | 36 | male | 18 | Memory deficit | Memory deficit | 7 | 125.5 | 3.36 | 0.54 | Abnormal signals in bilateral temporal lobe and hippocampus |
| 3 | 14 | male | 9 | Conscious disturbance, seizure | Cognitive dysfunction | 25 | 123.5 | 3.48 | 0.39 | Abnormal enhancement in bilateral temporal meninges |
| 4 | 24 | female | 11 | Conscious disturbance, psychiatric symptoms | Conscious disturbance, involuntary movement | 66 | 126.5 | 3.06 | 0.31 | Normal |
| 5 | 57 | male | 5 | Memory deficit, movement disorders | Memory deficit | 9 | 126.2 | 5.6 | 0.7 | Abnormal signals in right radial coronal area and bilateral temporal lobe |

Please find the attached excel files for Supplemental table 2/4-8.
